# Establishment of an endoplasmic reticulum stress-associated lncRNAs model to predict prognosis and immunological characteristics in hepatocellular carcinoma

**DOI:** 10.1371/journal.pone.0287724

**Published:** 2023-08-30

**Authors:** Xingyuan Shen, Siyuan Wu, Zhen Yang, Chunfu Zhu

**Affiliations:** 1 School of Graduate, Dalian Medical University, Dalian, Liaoning, China; 2 Department of General Surgery, The Affiliated Changzhou No.2 People’s Hospital of Nanjing Medical University, Changzhou, China; King Faisal Specialist Hospital and Research Center, SAUDI ARABIA

## Abstract

**Background:**

The endoplasmic reticulum stress (ERS) and unfolded protein response (UPR) pathways play an essential role in the pathophysiology of hepatocellular carcinoma (HCC), and activation of the UPR pathway is strongly associated with tumor growth. However, the function of ERS-associated long non-coding RNAs (lncRNAs) in HCC is less recognized.

**Methods:**

We have used TCGA (The Cancer Genome Atlas) to obtain clinical and transcriptome data for HCC patients and the GSEA (Gene Set Enrichment Analysis) molecular signature database to get the ERS gene. ERS-associated prognostic lncRNA was determined using univariate Cox regression study. Then, least absolute shrinkage and selection operator and multivariate Cox regression study were used to construct ERS-associated lncRNAs risk model. Next, we use Kaplan-Meier (KM) survival study, time-dependent receiver operating characteristic (ROC) curve, univariate and multivariate Cox regression study to validate and evaluate the risk model. GSEA reveals the underlying molecular mechanism of the risk model. In addition, differences in Immune cell Infiltration Study, half-maximal inhibitory concentration (IC50) and immune checkpoints blockade (ICB) treatment between high and low risk groups were analyzed.

**Results:**

We constructed a risk model consisting of 6 ERS-associated lncRNAS (containingMKLN1-AS, LINC01224, AL590705.3, AC008622.2, AC145207.5, and AC026412.3). The KM survival study showed that the prognosis of HCC patients in low-risk group was better than that in high-risk group. ROC study, univariate and multivariate Cox regression study showed that the risk model had good predictive power for HCC patients. Our verification sample verified the aforesaid findings. GSEA suggests that several tumor- and metabolism-related signaling pathways are associated with risk groups. Simultaneously, we discovered that the risk models may help in the treatment of ICB and the selection of chemotherapeutic drugs.

**Conclusions:**

In this article, we created an ERS-associated lncRNAs risk model to help prognostic diagnosis and personalized therapy in HCC.

## 1. Introduction

Hepatocellular carcinoma (HCC) is a frequent malignant tumor that occurs at a high rate in the population, particularly in locations where medical services are scarce. Such as, economically disadvantaged Asian countries and African countries [1]. The majority of people with HCC have pre-existing liver disorders, such as viral hepatitis, liver fibrosis, fatty liver or alcoholic liver disease [2],In the last decade, HCC Patients can benefit from surgical resection, liver transplantation, ablation, immunotherapy, and targeted therapy [3].Moreover, several HCC biomarkers, including blood and tissue biomarkers, are critical for the early detection and prognosis of individuals with HCC. However, certain biomarkers have the drawback of having limited sensitivity and specificity [4]. Hence, there is a need to develop reliable predictive risk assessment models for patients with HCC and seek accurate biomarkers to guide HCC therapy.

Long non-coding RNAs(lncRNAs) are among non-coding RNAs(ncRNAs), lncRNAs consisting of more than 200 nucleotides are engaged in several biological characteristics such as tumor growth and control of gene expression [5–7].With the progress of science and technology, microarray and transcriptome study have helped us find many lncRNAs that are associated with cancer [8, 9].while lncRNAs have been reported to have cancer-promoting and cancer-suppressing actions in malignancies(e.g., liver, breast, and colon cancers)among others [6]. Several lncRNAs have been confirmed to as biomarkers for detecting and treating HCC [8] (e.g.,ZFPM2-AS1 [10], LIN0572 [11] and SNHG17 [12]).Furthermore, lncRNAs take on a critical significance for immune cell activation, infiltration, and migration [13].

The endoplasmic reticulum (ER) refers to a vital cell apparatus for protein synthesis [14]. Endoplasmic reticulum stress (ERS) occurs when a number of risk factors (e.g., viruses, drugs, stress, hypoxia, poisoning and Ca2+ release) disrupt ER proteostasis, resulting in the deposition of misfolded/unfolded proteins in the ER. The unfolded protein response (UPR) is activated to reestablish ER proteostasis [15, 16]. Simultaneous activation of three sensors on ER membrane proteins consists of PKR-like ER kinase(PERK),activating transcription factor 6(ATF6),as well as inositol-requiring enzyme-1(IRE1) [16, 17]. If severe ERS occurs, the UPR signaling pathway will be activated for a long time, thus resulting in a disproportionate ratio of tumor suppressor genes to oncogenes, an elevated ratio of oncogenes, as well as the accelerated cancer progression [18]. Existing studies associated excessive ERS and UPR activation with the beginning and progression of a wide variety of illnesses(e.g., obesity, type 2 diabetes, atherosclerosis, neurodegenerative disorders, as well as malignant tumors) [19, 20].

Chronic ERS and sustained unfolded protein activation take on a critical significance for liver disease, and UPR has been found to have a correlation with liver inflammation, steatosis, hepatocyte apoptosis, hepatocyte autophagy, as well as liver fibrosis [15, 21, 22]. A number of ERS-associated proteins serve as biomarkers for HCC targeted therapy and prognostic diagnosis [23]. Therefore, the role played by ERS and sustained unfolded protein response on the liver should be studied in depth.

In this study, data relating to HCC-associated lncRNA expression and ERS genes were obtained according to databases. Bioinformatics was adopted for the identification of the risk model for lncRNAs correlated with ERS. It was coupled with clinical data, immunological checkpoints, immune infiltration and medication resistance for gaining insights into the diagnostic and prognostic utility of this model in terms of HCC in the future.

## 2. Methods

### 2.1. Information gathering and processing

RNA sequencing (RNA-seq) data were acquired from TCGA (The Cancer Genome Atlas)(https://portal.gdc.cancer.gov/) for 374 HCC samples and 50 adjacent normal samples, as well as clinical data from 378 HCC patients, which consisted of age, gender, futime, fustat, grade and TNM-stage. We excluded the clinical data with survival times less than 30 days because of the incompleteness of the data, as well as the clinical information with uncertain TNM-STAGE, futime, fustat, or grade. The collated clinical data were employed for subsequent study. Next, based on GSEA (Gene Set Enrichment Analysis) Molecular Signatures Database (https://www.gsea-msigdb.org/gsea/msigdb/index.jsp), We collected 37 ERS genes, which consisted of IRE1(M10426), PERK(M42776), and ATF6(M23457).

### 2.2. Collection endoplasmic reticulum stress-associated lncRNAs in hepatocellular carcinoma

First, Ensembl (http://ensemblgenomes.org/) offers the human.gtf file, and strawberry Perl was used to discriminate between mRNAs and lncRNAs in HCC tissues. After that, lncRNAs linked with ERS were identified using the “limma” package in R language, with the filtering settings CorFilter>0.4 and P-value<0.001. The ERS-associated lncRNAs co-expression network was built using 744 lncRNAs.

### 2.3. Establishment and verification of endoplasmic reticulum stress-associated lncRNAs prognostic risk model

We collected the clinical data of HCC patients obtained from TCGA, excluded those with a survival time of fewer than 30 days and uncertain survival status, and integrated it with TCGA transcriptome data, resulting in a total of 342 HCC samples with RNA sequencing data, survival time, and survival status. Meanwhile, the entire HCC sample was randomly divided into two groups for future study: training samples (n = 172) and verification samples (n = 170). Study of the training samples using univariate Cox regression (screen condition of P-value<0.01). The least absolute shrinkage and selection operator (Lasso)-Cox regression study was carried out using the R packages "survival," "caret," "glmnet," and "survminer" after the univariate regression study was completed. To avoid too many similar genes, the screening conditions were P-value<0.01 and 1000 cross-verification. Then, the ERS-associated lncRNAs with the most independent prognostic value were chosen for model construction utilizing multivariate Cox regression study. We scored the respective patient with HCC using a risk score equation and then assigned them into groups at a high risk and a low risk in accordance with their median risk scores.

Risk score equation = (lncRNA_1_ coefficient×lncRNA_1_ expression level) + (lncRNA_2_ coefficient ×lncRNA_2_ expression level) +…+ (lncRNAn coefficient×lncRNAn expression level). To evaluate the distinctions between the two groupings, we used the R language "survival" and "survminer" to conduct a Kaplan-Meier survival study. Moreover, the "timeROC" was used to accomplish the Receiver Operating Characteristic (ROC) curves at the 1, 3, and 5-year time points.

### 2.4. Evaluation of univariate and multivariate Cox regression study

Using univariate and multivariate Cox regression study to test whether the risk model we constructed can be used as an independent prognostic factor.(P-value<0.05 were statistically significant). Then, we use "timeROC" in R language which draws the multi-exponential ROC curve.

### 2.5. Enrichment functional and immune cell infiltration study

We utilized GSEA software to undertake functional and pathway enrichment analyses for groups at a high risk and a low risk to explore the possible biological mechanisms of our built risk model in depth. The gene sets database was selected cp.kegg.v7.4.symbols.gmt.NOM p-val<0.05 or FDR q-val <0.25 could meet the screening requirements of this study.

To download all TCGA tumor immune infiltration data, we utilized 7 different websites (CIBERSORT, CIBERSORTABS, TIMER, MCPCOUNTER, QUANTISEQ, EPIC and XCELL). Spearman correlation study was used to show the connection between the ERS-associated lncRNAs risk model and immune cell infiltration. Finally, the lollipop diagram is drawn using “ggplot2” in R language.

### 2.6. Evaluation of risk model about clinical chemotherapy drugs response and immune checkpoints

To assess the risk model sensitivity to different Chemotherapy drugs, the R package “pRRophetic” [24](https://github.com/paulgeeleher/pRRophetic)was employed for obtaining the half-maximal inhibitory concentration (IC50). A comparison was drawn in terms of the difference in immune checkpoint expression between the groups at a high risk and a low risk.

### 2.7. Consensus clusters study and evaluating tumor immune micro-environment (TME)

We use the “ConsensusClusterPlus” package in the R language. The objective of the ConsensusClusterPlus algorithm (CC algorithm) is to produce a stable molecular cluster by repeating a subsample of the data [25, 26].Clustering in accordance with the expression level of ERS-associated lncRNAs in HCC patient samples. The best clustering number was determined using the Empirical cumulative distribution function (CDF) plots. We further investigate the Kaplan-Meier survival study of various molecular subtypes and the correlation between different molecular subtypes in Tumor Immune Microenvironment (TME). Next, "Rtsne" package in R language was adopted for Principal component analysis (PCA).

### 2.8. Statistical study

For statistical study, this study made use of the R language (version 4.0.4) and the strawberry Perl language. The co-expression network of ERS genes and lncRNAs was built using the R package "igraph”. The Kruskal- Wallis test was used to compare the differences between the different groups. Each patient’s TME score was assessed using the "ESTIMATE" package, and the TME scores included the StromalScore, ImmuneScore, and ESTIMATEScore [27, 28]. KM survival curves were used to test whether there was a difference between the different groups. The "survival","survminer” and "timeROC" package is used to generate ROC curves and areas under curve (AUC). GSEA for functional study. Simultaneously, independent prognostic factors were investigated using univariate and multivariate Cox regression study. P-value<0.05 were considered statistically significant.

## 3. Results

### 3.1. Identification of endoplasmic reticulum stress-associated lncRNAs

**Fig 1:**
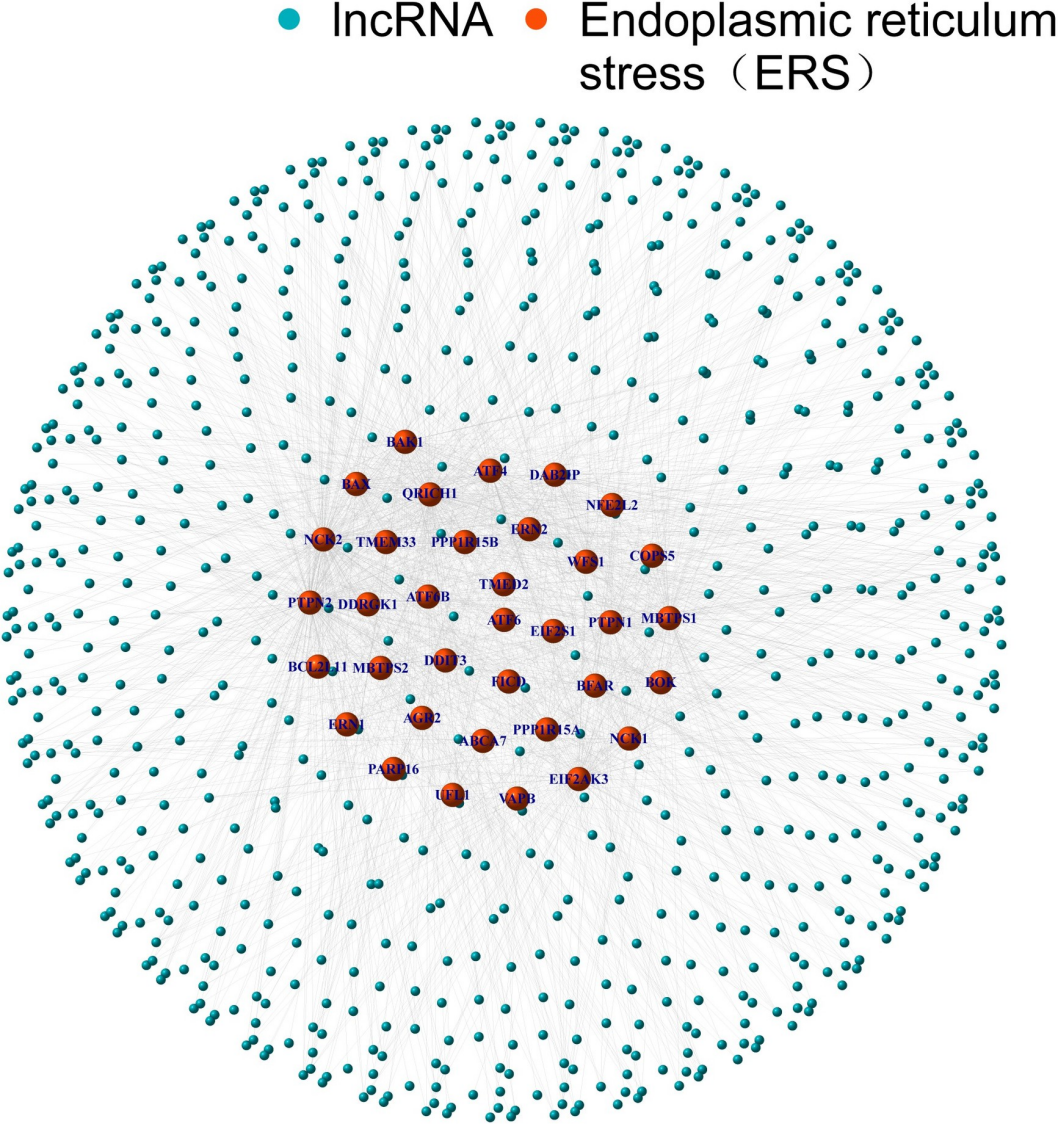


Firstly, we explored the Molecular Signatures Database for relevant ERS genes, including the IRE1, PERK, and ATF6 gene sets. According to the transcriptome data of TCGA, we identified 744 lncRNAs associated with ERS in total (CorFilter>0.4 and P-value<0.001), and draw co-expression network plot (Fig 1).

### 3.2. Six ERS-associated lncRNAs for establishing risk models

**Fig 2:**
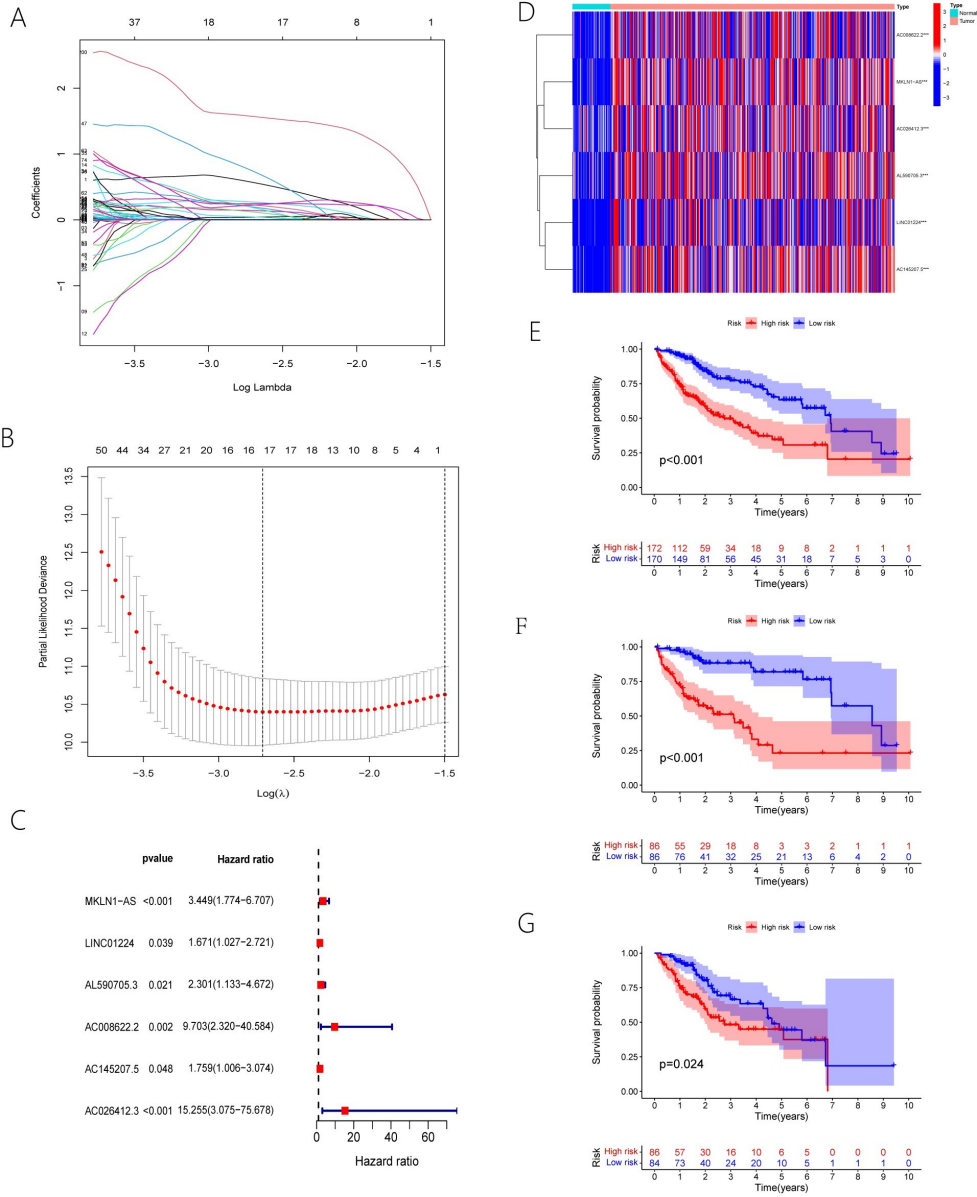


First,210 lncRNAs associated with prognosis were filtered out of the training samples using univariate Cox regression study (P-value< 0.01). The Lasso-Cox regression study was used for the lncRNAs mentioned before to prevent over-fitting genes. When Log(λ) is at its minimum value,16 lncRNAs can be used in the following multivariate cox regression study (Fig 2A and 2B). The final 6 lncRNAs were utilized to construct the risk model for ERS-associated lncRNAs and draw the forest plot(Fig 2C).When we evaluated the expression levels of the above six ERS-associated lncRNAs in HCC tumor and normal samples, we found that the above 6 ERS-associated lncRNAs expressed highly in HCC tumor samples(Fig 2D).The above 6 ERS-associated lncRNAs are MKLN1-AS,LINC01224, AL590705.3,AC008622.2,AC145207.5 and AC026412.3.The expression level and coefficient of the above six lncRNAs were used to calculate the risk score of each HCC patient, use the equation below.

Risk score equation = MKLN1-AS expression level×1.2381+LINC01224 expression level×0.5136+AL590705.3 expression level×0.8334+AC008622.2 expression level×2.2724+AC145207.5 expession level×0.5644+AC026412.3 expression level×2.7248.

The risk score equation was adopted for obtaining the risk score for the respective patient in the training samples and verification samples. Patients in the training samples were divided into groups at a high risk and a low risk in accordance with their median risk score. In addition, in accordance with median risk scores, patients in the verification sample were assigned into groups at a high risk and a low risk.

**Fig 3:**
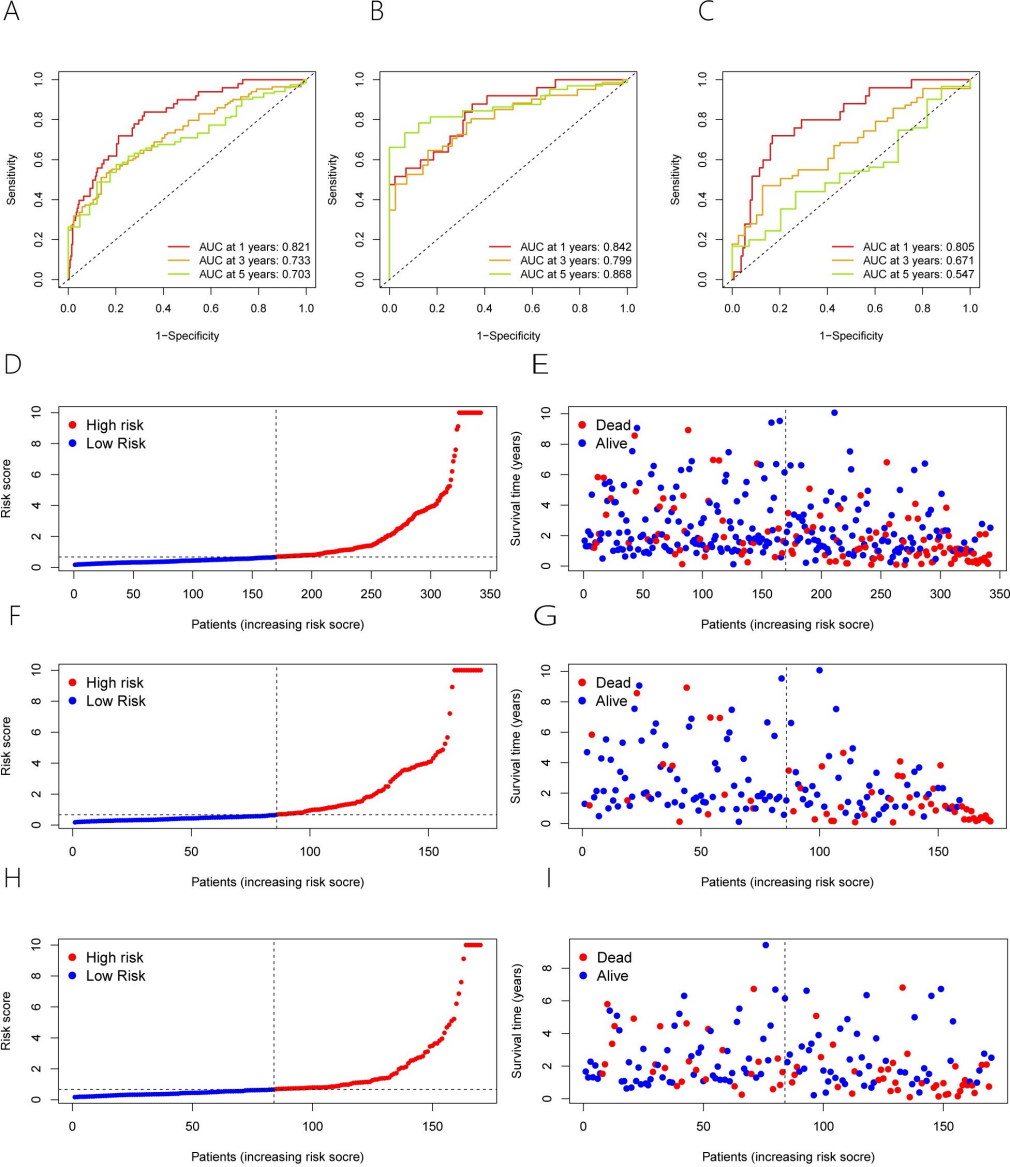


**Fig 4:**
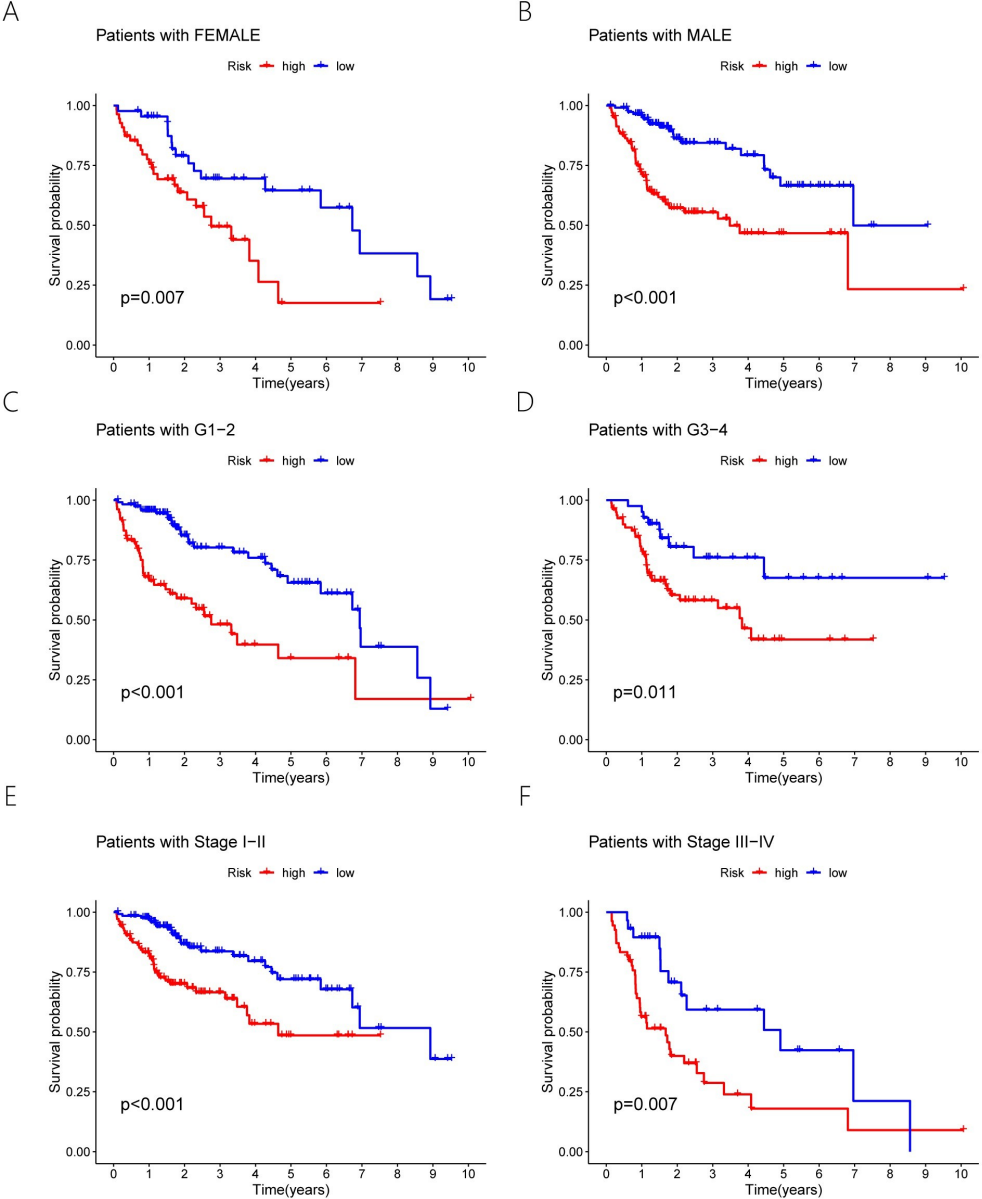


Following that, we performed Kaplan-Meier (KM) survival study on both the entire samples and training samples and discovered that overall survival was significantly shorter in the group at a high risk than in the group at a low risk (Fig 2E and 2F). In verification samples, overall survival was also shorter in the group at a high risk than in the group at a low risk (Fig 2G). Meanwhile, the three samples examined were submitted to a time-ROC curve study. The AUC for 1, 3, and 5 years was 0.821,0.733 and 0.703 in the entire sample and 0.842, 0.799 and 0.868 in the training sample, respectively (Fig 3A and 3B). Their AUC was 0.805,0.671 and 0.547 for 1, 3, and 5 years, respectively, in the verification samples (Fig 3C). Moreover, patient mortality increased as risk scores improved in all three samples (Fig 3D–3I). We stratified the different clinical features to further validate the risk model prognostic ability (such as gender, stage, and grade). In the entire samples, the group at a high risk also had a poorer prognosis across different clinical features (Fig 4A–4F). The above results suggest that 6 ERS-associated lncRNAs risk models have a better prognosis for patients with HCC.

### 3.3. Evaluating the independent prognosis of six ERS-associated lncRNAs risk models

**Fig 5:**
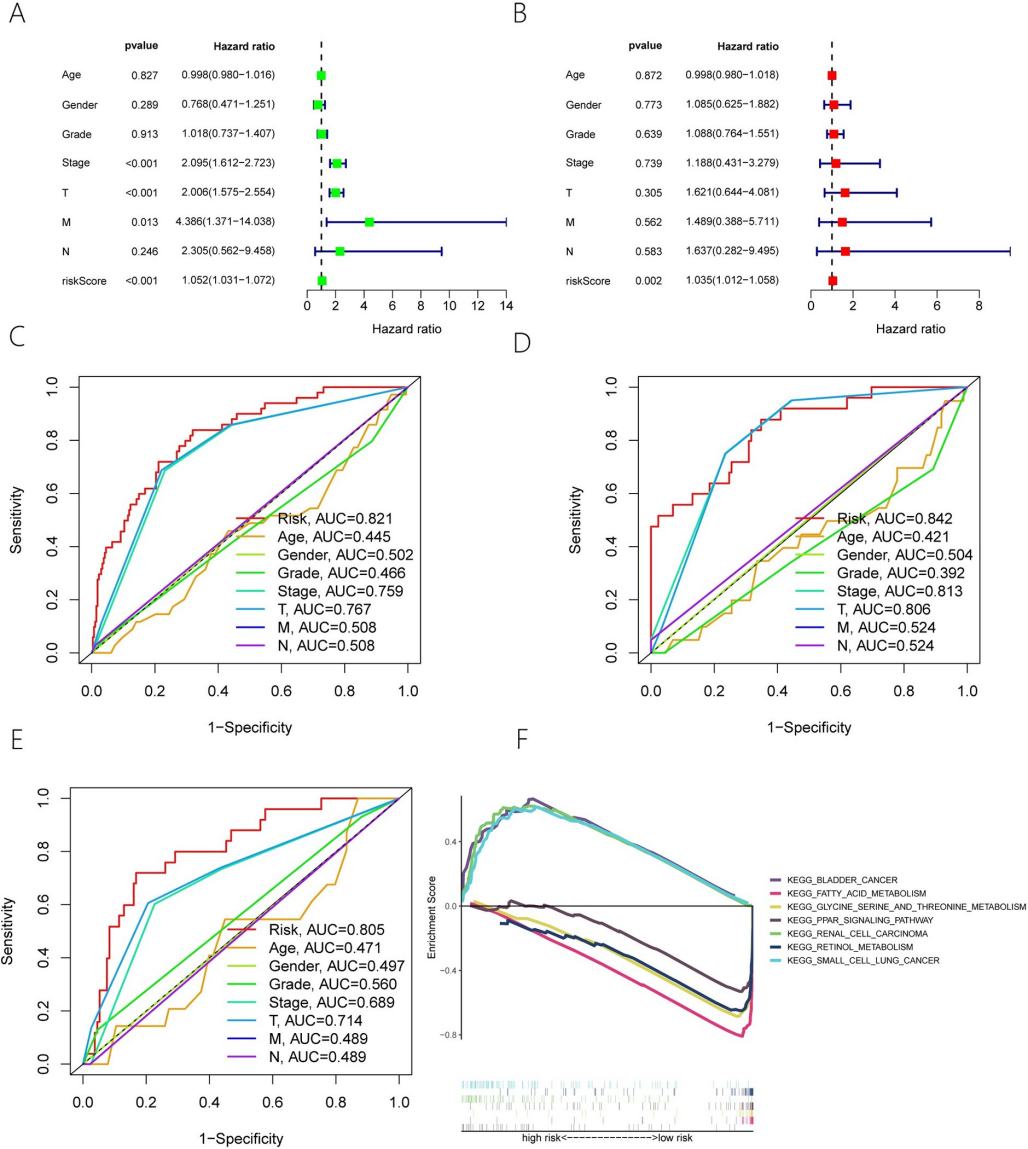


The Hazard ratio and 95% Confidence interval (CI) of the risk score for the entire samples were 1.052 and 1.031–1.072, in the univariate Cox regression study (p-value<0.001) (Fig 5A). The Hazard ratio and 95% CI of the Risk score for the entire samples were 1.035 and 1.012–1.058, respectively, in multivariate Cox regression study (P = 0.002) (Fig 5B). As a consequence of the above, we conclude that the risk model we developed may be utilized as an independent prognostic factor.

We subsequently performed multi-exponential ROC curve study on the risk scores of the entire samples, training samples and verification samples to investigate the correlation with different clinical features. The AUC for the three samples were 0.821,0.805 and 0.842 respectively (Fig 5C–5E). According to the multi-exponential ROC curve study, the AUC of the risk model was much better than the other clinical features.

### 3.4. GSEA (Gene set enrichment analysis)

In the entire HCC samples, the group at a high risk was enriched in DNA REPLICATION, RNA DEGRADATION, PATHWAYS IN CANCER, INSULIN SIGNALING PATHWAY.And the high risk is also enriched in BLADDER CANCER, SMALL CELL LUNG CANCER, RENAL CELL CARCINOMA. The group at a low risk was mainly enriched in GLYCINE SERINE AND THREONINE METABOLISM, PPAR SIGNALING PATHWAY, RETINOL METABOLISM, FATTY ACID METABOLISM (Fig 5F). The results of the aforementioned research further indicate that the risk model we created plays a different biological role in the development of cancers.

### 3.5. To investigate the link between immune checkpoints and chemotherapy drugs sensitivity in the ERS-associated lncRNAs risk model

**Fig 6:**
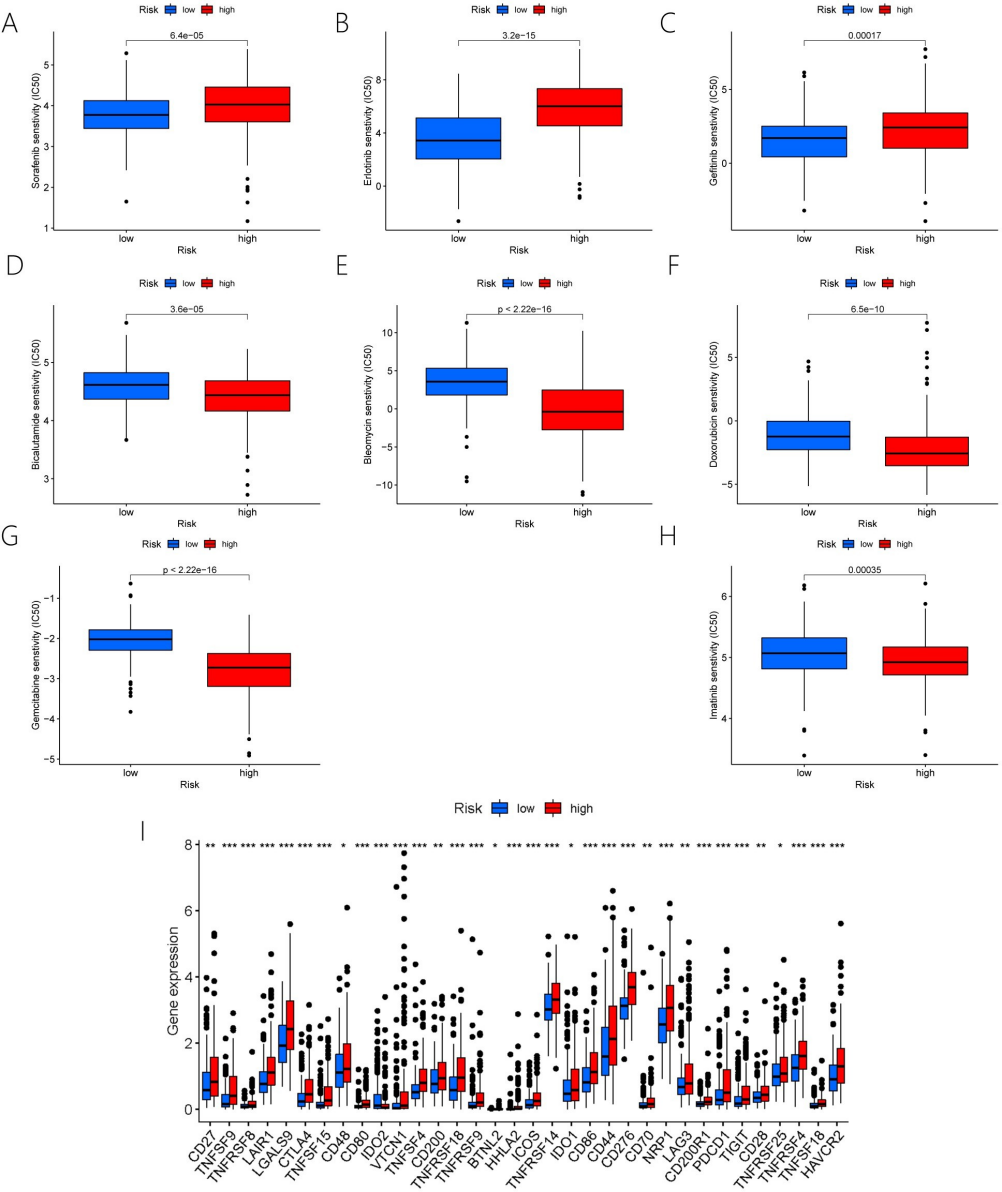


The pRRophetic package in R language was adopted for studying the sensitivity of groups at a high risk and a low risk to chemotherapies in the whole sample of HCC. As revealed by the high IC50 values of Sorafenib, Erlotinib, and Gefitinib in the group at a high risk, the group at a low risk had sensitivity to the above three medicines(Fig 6A–6C). In the group at a low risk, the high IC50 for Bicalutamide, Bleomycin, Doxorubicin, Gemcitabine, and Imatinib indicated that the group at a high risk was sensitive to the above five medications(Fig 6D–6H).Furthermore, this study suggested that the group at a high risk had more activation in 48 immune checkpoints(Fig 6I).

### 3.6. Correlation between ERS-associated lncRNAs risk model and immune cell infiltration

**Fig 7:**
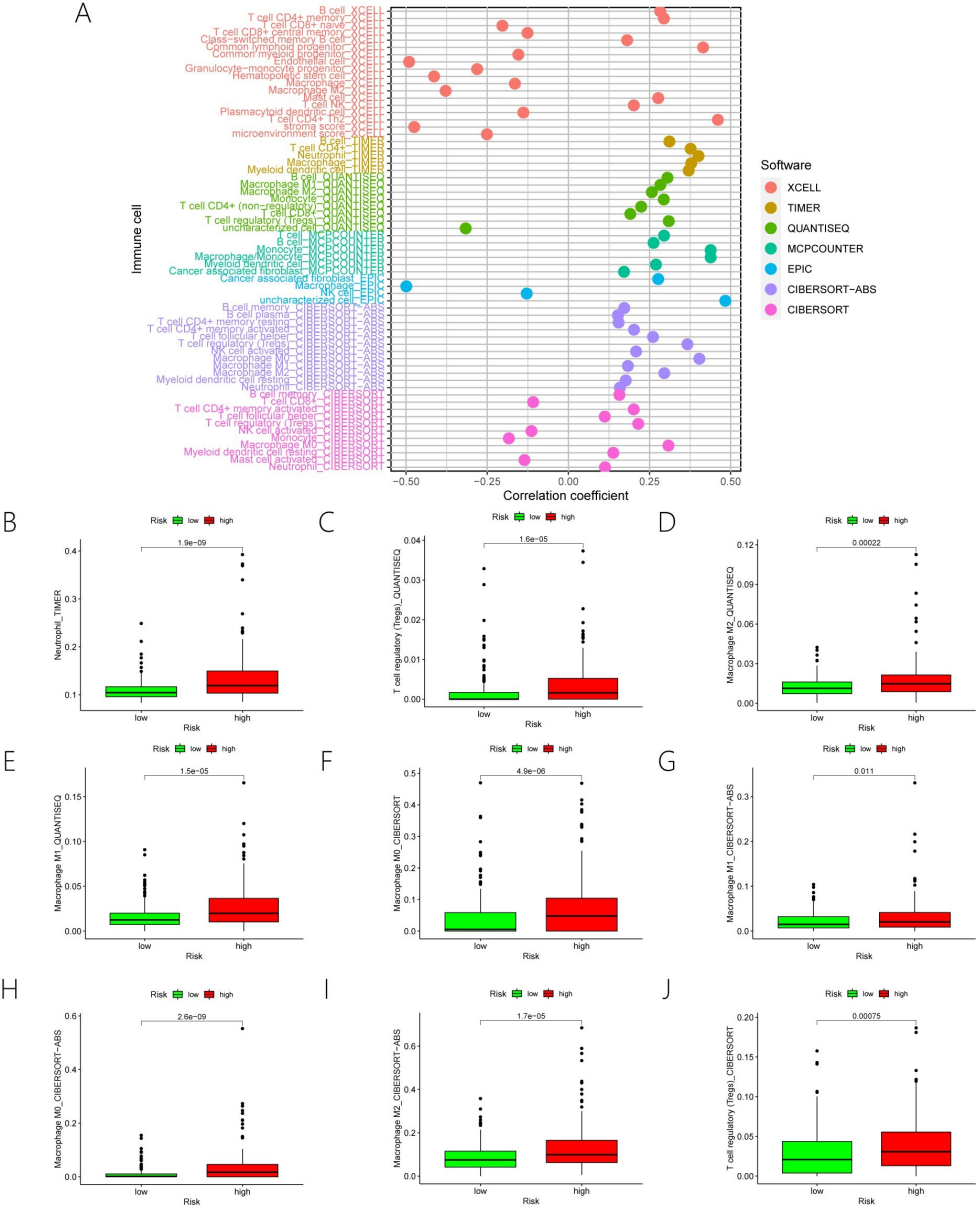


We found a connection between risk scores and immune cells across seven distinct platforms of immune cell infiltration. Such as, Neutrophil, Macrophage, T cell regulatory (Tregs), Macrophage M0, Macrophage M1, Macrophage M2, T cell CD4+ memory (Fig 7A). Further, the group at a high risk had a higher level of immune cell infiltration, which explains why patients in the group at a high risk had a worse prognosis (Fig 7B–7J).

### 3.7. Two molecular subtypes by consensus clustering algorithm

**Fig 8:**
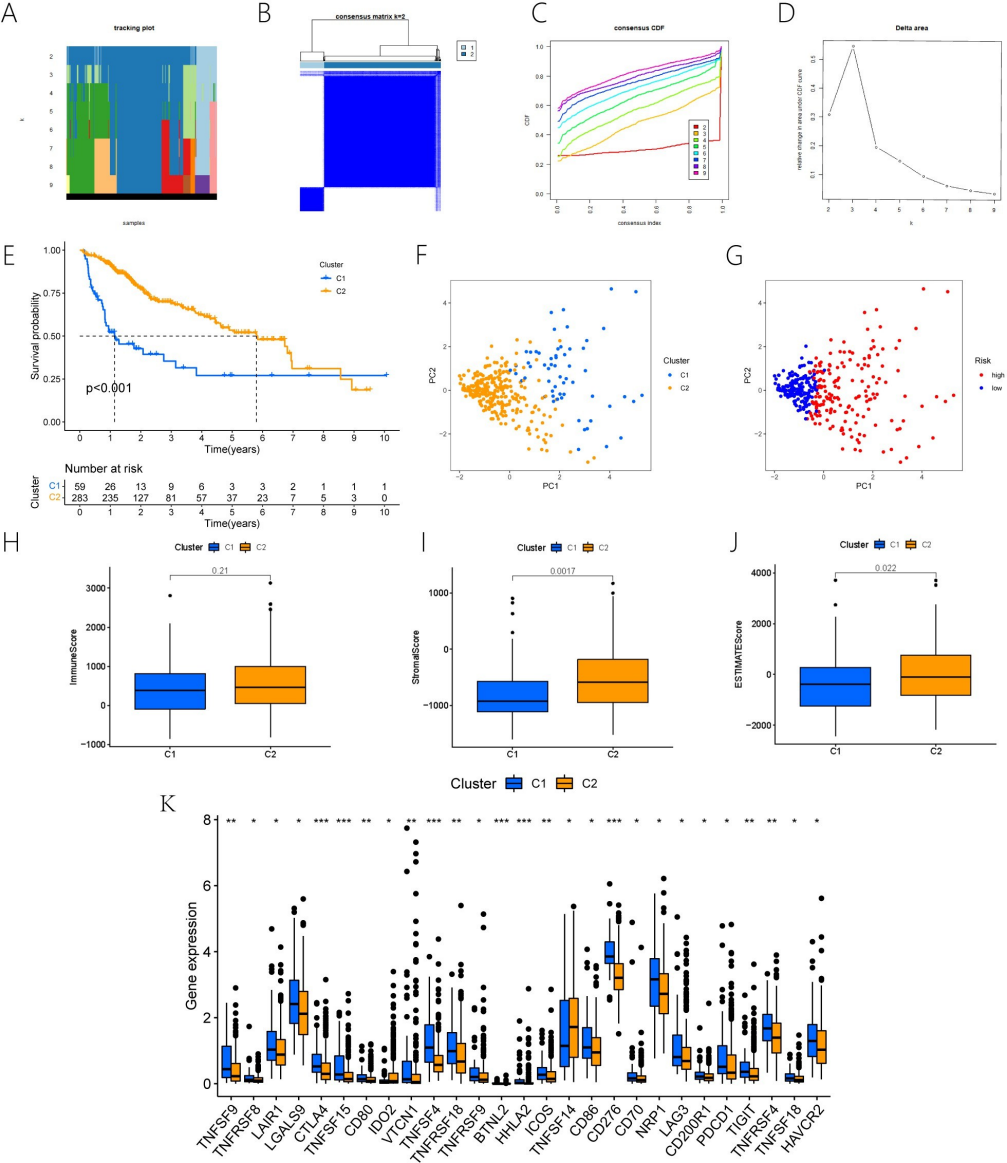


In accordance with the risk model we built for ERS-associated lncRNAs, consensus clustering was performed on the entire sample of HCC using the ConsensusClusterPlus algorithm. Different molecular subtypes are expressed by the consensus number (K) (k = 2–9) (Fig 8A). K = 2 (Fig 8B) is selected as the best molecular subtype in accordance with the cumulative distribution function (CDF) (Fig 8C) and relative change in area under the CDF curve (Fig 8D). As a result, the entire HCC sample was split into two molecular subtypes: Cluster 1 and Cluster 2. According to a KM survival study, the cluster 2 survival rate was significantly higher than Cluster 1(P-value<0.001) (Fig 8E). The principal component analysis (PCA) showed that Cluster 1 and Cluster 2 had different distribution areas, which means our grouping was correct (Fig 8F). Moreover, PCA study of the group at a high risk and a low risk achieved the same result (Fig 8G).

Then, Calculate the stromal, immune, and ESTIMATE scores of the Cluster 1 and Cluster 2 using the ESTIMATE algorithm. Although there was no significant difference in immune scores between Cluster 1 and Cluster 2 (P-value = 0.21) (Fig 8H), Cluster 1 and Cluster 2 exhibited different stromal (Fig 8I) and ESTIMATE scores(8J). Furthermore, we found that Cluster 1 had higher activation of 27 immune checkpoints (Fig 8k).

## 4. Discussion

Several clinical subtypes of primary liver cancer include HCC, intrahepatic cholangiocarcinoma, and others, but HCC accounts for approximately 80% of cases [29]. According to earlier research, lncRNAs have a significant biological function in HCC, and their aberrant expression may result in cell apoptosis, necrosis, dysregulation of cellular lipid metabolism, and increased oxidative stress burden [30]. Furthermore, we discovered that when the ER is stressed, genes linked with ERS establish a web of connections with lncRNAs that regulates tumor growth, including angiogenesis, immune infiltration, and micro-environment modification [16, 31]. In summary, we focused on lncRNAs associated with ERS and built a risk model to evaluate their influence on the prognosis and treatment of HCC.

In accordance with HCC patient clinical data and transcriptome data from the TCGA database,342 HCC patients were analyzed in this study. Co-expression study was used to find lncRNAs linked to ERS. We selected 6 ERS-associated lncRNAs to construct a risk model. Furthermore, KM survival study showed that patients with HCC in the group at a high risk survived less than those in the group at a low risk. Immune infiltration and immune checkpoint activation were also higher in the group at a high risk. The subsequent consensus clustering study also showed that Cluster 1 had a worse prognosis than Cluster 2. In the TME score, Cluster 1 and Cluster 2 had different TME scores, but Cluster 1 had more activation in the immune checkpoint. Furthermore, independent prognosis study revealed that the risk model we developed for ERS-associated lncRNAs was a credible independent prognostic factor. In entire samples, training samples, and verification samples, Multi-index ROC curve study and Time-dependent ROC curve study achieved prominent AUC values. In brief, the ERS-associated lncRNAs risk model could be credible. For the construction of the ERS-associated lncRNAs risk model, we identified six lncRNAs: MKLN1-AS, LINC01224, AL590705.3, AC008622.2, AC145207.5, and AC026412.3. Some of the above lncRNAs have important biological functions in HCC. MKLN1-AS has been linked to autophagy, pyroptosis, and hypoxia. In existing studies, high expression of MKLN1-AS decreases HCC patients disease-free survival (DFS) and overall survival (OS). MKLN1-AS oncogenic involvement in cancer is linked to YAP1,and MKLN1-AS can increase HCC development by inducing YAP1 expression [32]. LINC01224 promotes the growth of different malignancies. LINC01224 binds to miR-330-5p and inhibits the development of HCC by having an effect on the expression of the downstream target gene CHEK1 [33]. Furthermore, clinical risk models in accordance with AC145207.5 and AC008622.2 have been built for better clinical diagnosis [34, 35]. Even though AL590705.3 and AC026412.3 have been investigated less in HCC, their correlation with malignancies should be elucidated.

In this study, the risk score was significantly correlated with immune infiltration. Tregs, Macrophage M0, Macrophage M1, Macrophage M2, and Neutrophil were found to be infiltrated in the group at a high risk of HCC patients. As reported by existing research, neutrophils create cytokines and chemokines in tumors, thus recruiting macrophages and Tregs and having an effect on HCC progression and prognosis [36]. Tumor-associated macrophages (TAMs) are of great significance to the tumor micro-environment (TME), and M2 macrophages significantly affect tumor occurrence and progression. The release of IL-4 and TGF-β by M2 macrophages inhibit the growth of NK cells and T cells, thus resulting in reduced anti-tumor immunity in the host and insensitivity to chemotherapeutic treatments [37, 38]. Besides, Tregs expression is correlated with a poor prognosis in cancer patients [39]. Tregs suppressed anti-tumor immune responses by interacting with fibroblasts and endothelial cells [40]. Anti-cytotoxic T lymphocyte-associated antigen-4 (CTLA-4) antibodies, on the other hand, have shaped novel anti-tumor treatment, and they can deplete Tregs in the tumor micro-environment, so they show a novel direction of targeted cancer therapy [41, 42].CD8+ T cells were found to increase in patients treated with anti-PD-1 and anti-PD-L1 antibodies. As revealed by the increase in CD8+ T cells, anti-PD-1 therapy could be effective against the tumor, and CD8+ T cells could contribute to tumor cell death [43, 44].Consequently, we conclude that HCC patients with more immune cell infiltration can be better selected for immunotherapy. Immune checkpoint blockade (ICB) in tumor treatment have attracted increasing attention in recent years. The group at a high risk showed more significant levels of immune checkpoints in our risk model. The group at a high risk had significantly higher levels of LAG3, TNFRSF14, TNFRSF18, TNFRSF9, TNFRSF8, TNFRSF18, CTLA4 and TIGIT expressions as compared with the group at a low risk. Numerous members of the Tumor Necrosis Factor Receptor Superfamily (TNFRSF) are required stimulatory signals creating costimulatory domains with CAR-T cells to improve CAR-T cell treatment for patients [45]. Monoclonal antibodies (mAbs) are capable of suppressing TIGIT, thus hindering the development of multiple myeloma (MM) [46]. As revealed by the consensus clustering study, even though the overall survival (OS) of Cluster 1 was poor, Cluster 1 could receive more ICB treatment, which would facilitate the prognosis of Cluster 1 patients. As revealed by the results of this study, the risk model we built could provide a reference for future immunotherapy and ICB treatment.

Chemotherapy drugs have been extensively employed for treating advanced HCC patients, whereas high drug resistance has an effect on their prognosis [47, 48]. Our analyses suggested that patients with high-risk HCC might consider using Bicalutamide, Bleomycin, Doxorubicin, Gemcitabine, and Imatinib. Patients with low-risk HCC. On the other hand, are better candidates for Sorafenib, Erlotinib, and Gefitinib. Targeting the ERS and UPR pathways is beneficial for cancer treatment. PERK/ATF4 pathway inhibitors reduce resistance to sorafenib in HCC patients [49]. Doxorubicin cooperates with PERK pathway to activate autophagy in hepatoma cells [50]. Although this is only a bioinformatics study, it also provides a reference for the use of chemotherapy drugs.

This study has some limitations. First, this study used a public database instead of a sample of its own, and the data in the database was incomplete. The training samples and the test samples originated from the same database. Second, we simply conducted bioinformatics study without performing further cell and animal research to confirm our findings, especially the correlation of chemotherapeutic drugs and risk models.

## 5. Conclusions

In this study, the six ERS-associated lncRNAs identified were adopted to build a reliable, independent and valuable predictive risk model. This risk model could be used to more effectively made a prognosis of HCC patients and could be a potential biomarker for immunotherapy targets. In subsequent research, the risk model offers novel perspectives and references on personalized therapy of HCC.
